# Topics of the Month

**Published:** 1896-04

**Authors:** 


### TOPICS OF THE MONTH.

A TYPHOID fever epidemic at Elmira has been reported with a somewhat startling death-roll. It is asserted that the sewage of a number of towns and cities is poured into the Chemung river, which can have only one effect—namely, that of polluting and poisoning its water. Ice, too, is cut from that river and used at Elmira. It is a well-known fact that freezing does not destroy the poisonous germs of ice made from polluted water. We assume that here are ample causes for this outbreak of an infectious disease, which is recognised as preventable.

The prompt action of the health department in Buffalo has served to check a threatened epidemic through infected milk. A milk dealer was detected in delivering infected milk. He was prevented from further service to his customers and so the spreading of the disease was cut short. Had the health department of Elmira taken similar precautions, sickness and death to an alarming degree might have been prevented. These two lessons of cause and effect—of infection and prevention—teach their own moral.

---

IT IS reported that physicians in New York are finding it convenient to establish downtown offices in the large office buildings that are springing up in the business section. The argument in part is, that it divorces business from home interests, a condition of things never possible with office and residence under the same roof. In this day and age of specialties it may be practicable in many instances to establish this custom, particularly in great cities like New York, Chicago and Philadelphia. Nevertheless, it must ever remain as a well-established fact, that it is better for the general practiser of medicine, the family doctor, to keep his office and house together. He is often wanted in an emergency when time would be lost in reaching him through the uncertainty attending his whereabouts as between house and office, were they remotely separated. The young physician may start his professional career downtown amid the business whirl, but the well-established doctor will feel more in keeping with his calling to receive and serve his patients from the quieter surroundings of a residence population.

---

THE rapid strides that chemistry has recently made is a subject of much comment among those most familiar with its marvelous progress. The fact is, chemistry is the base of all the sciences, and to it should be accredited such of the progress in medicine that is thoughtlessly bestowed elsewhere. Chemistry has done more to relieve the medical art of the opprobrium of nauseous dosage than any other and all influences combined. It extracts juices, alkaloids and other concentrated principles from crude mineral and vegetable sources, and serves them up to us in minute doses that are both potent and agreeable.

Synthetic chemistry, too, is a marvel of scientific accomplishment and is destined to become as useful as it is curious. It not only produces many useful drugs of the antipyretic and hypnotic series from coal tar, but it has lately turned its attention to the production of artificial musk from the same source. Though this is not chemically the same as real musk, its scent is undistinguishable from the latter, and it threatens to drive the real article out of the market. One of the greatest commercial triumphs in the way of an artificial flavoring is vanalline. This product is keeping down the price of vanilla beans, and it is likely, too, to drive the latter out of the market. Chemists know how to counterfeit lactic acid, and they make an artificial citric acid which cannot be detected from the sour of the lemon. It is hardly possible to determine what may be the ultimate results of these counterfeiting processes, but they are somewhat startling to contemplate and furnish a subject for serious thought.

---

The milk supply of a large city is always an important subject for study and discussion by the guardians of public health. It is one that has been very much in evidence in Buffalo for some time past. Nearly a year ago the Academy of Medicine, through Dr. Henry R. Hopkins, chairman of sub-committee, presented an elaborate and well-digested report on the subject that has attracted the attention of sanitarians throughout the country. Meanwhile, a number of prominent physicians have inaugurated a plan of supplying pure milk to such residents of Buffalo as may desire to purchase it. The physicians referred to are Drs. Irving M. Snow, H. R. Hopkins, DeLancey Rochester, Charles G. Stockton and Renwick R. Ross. They have effected an agreement with Mr. George D. Briggs, of Elma, N. Y., who is authorised to sell milk produced at his farm under the name of certified milk. In the contract with Mr. Briggs, he engages to put upon the market only pure milk from high-grade Jerseys, free from tuberculosis. He also undertakes that the cows shall be fed on the best grass and hay.

The water supplied to the cattle has been certified to by Prof. Herbert M. Hill, chemist. The appointments of the farm are as perfect as sanitary science can make them, and each bottle of milk supplied will be marked with the day and hour of milking. The product will be submitted to a bacteriological examination every two weeks and the cows will be inspected every month by Dr. John Wende, V. S.

The physicians referred to have no pecuniary interest whatever in the undertaking, their sole purpose being to procure for their patients, and for the public generally, the proper kind of milk food.

Orders for certified milk may be left with Mr. C. W. Boyce, grocer, corner of Main and Allen streets.

---

THE New York board of health, it is alleged, (*Buffalo Morning Express*, March 16, 1896,) is the first body of that kind to attempt to apply the theory of the cathode rays to the killing of the bacilli of certain diseases. During that week Dr. Herman Biggs and Dr. A. L. Beebe of the bacteriological department, and Dr. E. W. Martin, the chief inspector of the food division, conducted a series of experiments in this new field of research. Last week the necessary apparatus for producing the rays, consisting of a Crooke's tube and a Ruhmkorff coil, was set up in the chemical laboratory of the board. As a sort of preliminary investigation, the bacilli of diphtheria were exposed to the rays for an hour and a half, and afterward were returned to the incubator for the purpose of observing the effect. On examination, it was found that the bacilli showed signs of growth, and that, consequently, the bacilli were not harmed by the new light. It was explained, however, that the experiment was a very rough and crude one, and that too much significance should not be attached to it. The general theory of the killing of bacilli in this way is based upon the fact that too much light is fatal. It is reasoned that the extreme light of the cathode rays ought to be especially destructive.

There are bacilli, not to say parasites, that are affecting the body politic that are also afraid of the effect of extreme light—even the broad sunlight of publicity—as Mr. Henry Watterson so graphically pointed out some years ago.

---

A NUMBER of clergymen in New York City, at a recent meeting, adopted a resolution appealing to the members of their respective congregations to relieve them from the attendance at cemeteries after performing the usual funeral rites. They allege, and with much reason, that after a long ride and exposure to all sorts of weather they are liable to contract pneumonia and other diseases, which may be prevented if the funeral ceremonies are completed at the church, house or other appropriately appointed place. The following extract from the appeal presents the subject in a very considerate and conservative light:

We might question the whole matter of interment services, on the ground of their extreme trial to the feelings and, in our climate, their danger to the health of those who attend them. For fully one-half the year the severities of our climate make the exposures they involve perilous to the witnesses and to the clergymen alike. The voice of the medical fraternity has repeatedly been raised against them, and there are not a few in every church who bear in memory some fatal illness whose seeds were sown at the interment of some friend. It seems at once useless and wrong to make the respect we would show the dead put the living in danger.

For a long time we have been of the opinion that some reform should be inaugurated in this matter. The appeal very appropriately affirms that it is useless and wrong to pay respect to the dead that in any manner places the life or health of the living in jeopardy.

---

THE friends of medical education were pleased to learn through these columns last month that the so-called Stanchfield bill, introduced in the legislature in the interests of those opposed to the present methods of medical education, had received such opposition as to practically arrest its further progress. They were equally pleased to learn through the newspaper press that the bill introduced by the Hon. Mr. Nussbaum, of Albany, had passed the senate and would probably meet no opposition in the assembly. Later, however, they were dismayed to learn that Mr. Edward Lauterbach, Mr. Platt's chairman of the republican county committee in New York City, had held up the Nussbaum bill in the assembly. He has been employed by those opposed to the measure, who are in reality the proponents of the Stanchfield bill, to prevent its passage.

Here surely is a nice state of affairs, for it is not difficult to discern in it a Platt-Tammany combination to prevent reform measures relating to medical education from passing the legislature. The enlightened medical profession, without regard to political preferences, will not be slow to resent the insult that has been so ruthlessly put upon it. It is just possible that this combination of all that is depraved in politics will soon witness the verification of the proverb that "whom the gods would destroy they first make mad."

LATER.—It is pleasant to inform our readers, however, that in spite of the action of Mr. Lauterbach, the Nussbaum bill was passed by the assembly March 20th, and that Governor Morton signed it on the 21st, so that it is now safely engrossed on the statute book. Among other things it prescribes a four years' course after January, 1898.

---

FRENCH physicians (*Lehigh Valley Medical Magazine*) are complaining, first, that there are too few foreign medical students coming to her hospitals for instructions and, secondly, far too many foreign physicians, many of them elderly, are practising medicine in their country. One of their medical societies recently discussed the dual question of how to increase the number of foreign students and decrease the number of foreign practitioners of medicine.

